# Genome-wide DNA methylation profiling reveals novel epigenetically regulated genes and non-coding RNAs in human testicular cancer

**DOI:** 10.1038/sj.bjc.6605505

**Published:** 2010-01-05

**Authors:** H H Cheung, T L Lee, A J Davis, D H Taft, O M Rennert, W Y Chan

**Affiliations:** 1 Laboratory of Clinical Genomics, Section on Developmental Genomics, Eunice Kennedy Shriver National Institute of Child Health and Human Development, National Institutes of Health, Bethesda, MD 20892, USA; 2 School of Biomedical Sciences, the Chinese University of Hong Kong, H.K.S.A.R., China

**Keywords:** DNA methylation, MeDIP-chip, non-coding RNA, intergenic and intronic DMR, TGCT

## Abstract

**Background:**

Testicular germ cell tumour (TGCT) is the most common malignant tumour in young males. Although aberrant DNA methylation is implicated in the pathophysiology of many cancers, only a limited number of genes are known to be epigenetically changed in TGCT. This report documents the genome-wide analysis of differential methylation in an *in vitro* model culture system. Interesting genes were validated in TGCT patient samples.

**Methods:**

In this study, we used methylated DNA immunoprecipitation (MeDIP) and whole-genome tiling arrays to identify differentially methylated regions (DMRs).

**Results:**

We identified 35 208 DMRs. However, only a small number of DMRs mapped to promoters. A genome-wide analysis of gene expression revealed a group of differentially expressed genes that were regulated by DNA methylation. We identified several candidate genes, including *APOLD1*, *PCDH10* and *RGAG1*, which were dysregulated in TGCT patient samples. Surprisingly, *APOLD1* had previously been mapped to the TGCT susceptibility locus at 12p13.1, suggesting that it may be important in TGCT pathogenesis. We also observed aberrant methylation in the loci of some non-coding RNAs (ncRNAs). One of the ncRNAs, hsa-mir-199a, was downregulated in TGCT patient samples, and also in our *in vitro* model culture system.

**Conclusion:**

This report is the first application of MeDIP-chip for identifying epigenetically regulated genes and ncRNAs in TGCT. We also demonstrated the function of intergenic and intronic DMRs in the regulation of ncRNAs.

Testicular germ cell tumour (TGCT) is an invasive germ cell tumour histologically classified as seminoma and non-seminoma. Non-seminomatous tumours can be further sub-classified into embryonal carcinoma, teratoma, choriocarcinoma and yolk-sac tumour. Most non-seminomatous tumours include multiple cell types. Embryonal carcinoma is the most frequent non-seminomatous tumour. It represents ∼87% of non-seminoma (Bosl and Motzer, 1997). Few seminomatous cell lines have been identified to date; several embryonal carcinoma cell lines have been established and shown to be useful for pathobiological and clinical studies (Andrews *et al*, 2005). Ntera2 (NT2) is one of the established pluripotent human testicular embryonal carcinoma cell lines. This cell line has been extensively used in research on TGCT (Burger *et al*, 1998; Koch *et al*, 2003; Skotheim *et al*, 2005). In this study, we used NT2 as a cell model to study differential methylation in embryonal carcinoma.

Unlike many cancers that peak during old age, TGCT is common in young males. Risk factors include cryptorchidism, prenatal exposure to diethylstilbestrol and genetic factors, such as locus Xq27 that increases susceptibility to develop TGCT (Rapley *et al*, 2000; Horwich *et al*, 2006). DNA mutation may be one of the causes of TGCT; however, accumulating information suggests a more prominent role for epigenetic alteration as a factor in tumourigenesis, including TGCT (Feinberg *et al*, 2006; Esteller, 2007). Previous reports on aberrant methylation of tumour-suppressor genes/oncogenes provide information of an epigenetic role in tumour development. Many studies focused on individual target genes. The first genome-wide study of DNA methylation in TGCT used the technique of restriction landmark genome scanning (Smiraglia *et al*, 2002). However, no report of global high-resolution analysis of methylation changes in TGCT has been published. Tiling array technology permits the elucidation of differentially methylated regions (DMRs) of the whole genome (Weber *et al*, 2005; Zhang *et al*, 2006; Cokus *et al*, 2008) by the ChIP-chip approach. A popular ChIP-chip-based method is methylated DNA immunoprecipitation (MeDIP), in which methylated DNA is enriched by the use of antibodies directed against 5-methylcytidine and hybridised to custom arrays, such as promoter arrays or CpG island microarrays (Yan *et al*, 2002; Jacinto *et al*, 2007; Irizarry *et al*, 2008). These whole-genome approaches are powerful tools for the identification of differentially methylated genes that may be important in tumourigenesis.

In this report, we used MeDIP, in combination with human tiling microarrays (MeDIP-chip) that allow coverage of the entire human genome, to elucidate DMRs. This approach allows the identification of not only differentially methylated promoters and gene-associated CpG islands but also differentially methylated non-coding RNAs (ncRNAs), such as microRNAs (miRNAs). An increasing number of reports suggest that miRNAs may have pivotal roles in tumour progression and development, including the regulation of neoplastic transformation and metastasis (Ma *et al*, 2007; Huang *et al*, 2008; Varambally *et al*, 2008). Some miRNAs are epigenetically silenced in cancer cells as a result of cancer-specific hypermethylation (Han *et al*, 2007; Lujambio *et al*, 2008; Toyota *et al*, 2008). As most miRNAs are located in intergenic or intronic regions, they were not identified in previous studies using promoter or CpG island arrays. To validate the clinical usefulness of our approach, we documented methylation and expression changes of three novel genes and a miRNA in a normal and tumourous testicular tissue. Our genome-wide approach demonstrates the use of MeDIP-chip integrated with expression profiling as a tool for identifying methylation-regulated genes and ncRNAs that might be important in disease.

## Materials and methods

### Primary tumour specimens, cell cultures and drug treatment

Genomic DNA (17 cases) and RNA (18 cases) samples obtained from TGCT patients were purchased from Oncomatrix (San Marcos, CA, USA). Normal testicular DNA (6 cases) and RNA (8 cases) were purchased from Biochain (Hayward, CA, USA) and Zyagen (San Diego, CA, USA), respectively. RNAs of tumour and normal adjacent tissues of other tumour types were purchased from Ambion (Austin, TX, USA). Each RNA sample was isolated from a single individual. Cell culture system Ntera2 (NT2), Tera-1 and normal human testis cell line CRL-7002 (HT) were purchased from ATCC (Manassas, VA, USA) and cultured in DMEM (Invitrogen, Carlsbad, CA, USA) supplemented with 10% FBS and incubated in a 37°C humidified incubator supplied with 5% CO_2_. For demethylation analysis, 1 × 10^5^ NT2 cells were seeded for 24 h and treated with 1–5 *μ*M of 5-aza-2-deoxycytidine (Sigma, St Louis, MO, USA) for 72 h.

### MeDIP and microarray hybridisation

Methylated DNA immunoprecipitation was performed as described previously (Weber *et al*, 2005). Briefly, genomic DNA was sheared by sonication on ice to generate random fragments of 100–500 bp. Sonicated DNA of 5 *μ*g was used for IP. Heat-denatured DNA was incubated with 10 *μ*l of mouse anti-5-methylcytidine monoclonal antibody (Eurogenetec, San Diego, CA, USA) in 1 × IP buffer (10 mM Na-Phosphate pH 7.0, 140 mM NaCl and 0.05% Triton X-100) with periodic shaking for 2 h at 4°C. Sheep anti-mouse IgG-conjugated Dynabeads (Invitrogen) were added to the IP buffer and incubated for an additional 2 h. The beads were washed thrice with 700 *μ*l 1 × IP buffer and then resuspended in 250 *μ*l digestion buffer (50 mM Tris, pH 8.0, 10 mM EDTA, 0.5% SDS). The antibodies were digested with 80 *μ*g of proteinase-K for 3 h at 50°C. DNA was extracted with phenol–chloroform and precipitated with ethanol. Precipitated DNA was resuspended in water and used for real-time quantitative (qPCR) (for validation of IP efficiency) or for microarray hybridisation. Several positive and negative control loci were used for confirmation of IP efficiency before hybridising to microarrays (Supplementary Figure 1A). The immunoprecipitated DNA was amplified, labelled and hybridised to Human Tiling Array 2.0R Chips (Affymetrix, Santa Clara, CA, USA) sequentially, as suggested by Affymetrix ChIP-chip protocol. Triplicate sets of hybridisation were performed from three independent MeDIP experiments for each cell line. Both tiling and expression arrays were washed and stained on the Affymetrix Fluidic Station 450 and Chips were scanned on GeneChip Scanner GCS3000 (Affymetrix).

### Tiling array data analysis

Raw CEL data files obtained from tiling array experiments were analysed using Tiling Analysis Software (TAS) (Affymetrix). Arrays from each group (cancer *vs* normal) were quantile normalised, and differential methylation between cancer and normal groups was compared by choosing the ‘two-sample comparison analysis’ option in TAS. A two-sided test was conducted to evaluate both hypermethylation and hypomethylation. A bandwidth was set at 275, such that the sliding window (2 × bandwidth+1) of the analysis is 551. Transfrags (or DMRs) were generated by interval analysis with a *P*-value cutoff at 20 (*P*<0.01), maximum gap at 250 and a minimum run at 50. Transfrags generated by the *P*-value cutoff with a positive signal difference were defined as hypermethylated, whereas those of negative difference were defined as hypomethylated. Genomic bisulphite sequencing was performed to confirm the sensitivity of the observed DMRs (Supplementary Figure 1C). Mapping of DMRs to Refseq, CpG island, promoter, miRNA and small nucleolar RNA (snoRNA) was performed using the Table Browser function embedded in UCSC Genome Bioinformatics (Santa Cruz, CA, USA; http://genome.ucsc.edu/cgi-bin/hgTables?command=start) or by our customised web-based tool TileMapper (http://tilemapper.nichd.nih.gov/tilemapper) designed specifically for transfrag mapping. Promoter annotation was retrieved from Genomatix (San Jose, CA, USA; http://www.genomatix.de), and the coordination of each promoter was stored in BED files. Annotations of Refseq, CpG island, miRNA and snoRNA were retrieved from the UCSC Genome Browser. All analyses were based on human genome Build 35.1.

### Expression array hybridisation and data analysis

Total RNA was extracted from NT2 and HT cells with Trizol reagent and analysed using Bioanalyzer (Agilent, Santa Clara, CA, USA). DNaseI-treated RNA of 3 *μ*g was amplified and the resulting cRNA was biotin-labelled and hybridised to Human Genome U133 Plus 2.0 Arrays (Affymetrix). Triplicate sets of hybridisation were performed for each cell line, and the raw data were normalised by robust multiarray average algorithm and analysed in Partek Genomics Suite Software (St Louis, MO, USA). Differential gene expression was evaluated using one-way ANOVA. Expression fold change of differentially methylated genes was represented by probing the most significant *P*-value. Differentially expressed genes were confirmed by real-time PCR (Supplementary Table 3).

### Genomic bisulphite sequencing and methylation-specific PCR

Genomic DNA (400 ng) was treated with sodium bisulphite using the EZ DNA Methylation-Gold Kit (Zymo Research, Orange, CA, USA). Bisulphite-treated DNA (80–100 ng) was used for PCR amplification. For bisulphite sequencing, the PCR product was TOPO-cloned into the pCR4 vector (Invitrogen) and 5–10 positive clones were sequenced. Graphics of CpG methylation were generated by CpGviewer (Leeds, UK; Carr *et al*, 2007). For methylation-specific PCR (MSP), methylated- and unmethylated-specific primers were designed in the same genomic region as in bisulphite sequencing. Methylation-specific PCR products were resolved in 2.5% agarose gel.

### Quantitative real-time RT–PCR

Total RNA (1 *μ*g) was primed by random hexamers and converted into cDNA by SuperScript III (Invitrogen). The SYBR green-based real-time PCR was performed in an Applied Biosystems 7500 Fast Real-Time PCR System (Applied Biosystems, Foster City, CA, USA), and the level of gene expression was normalised by 18S rRNA. For real-time quantification of miRNAs, total RNA was extracted using the mirVana miRNA Isolation Kit (Ambion). cDNA was synthesised from 1 *μ*g of total RNA using miRNA-specific primers with the TaqMan MicroRNA Reverse Transcription Kit (Applied Biosystems) and normalised by hsa-mir-191. All PCR primers are listed in Supplementary Table 4.

### Statistical analysis

The *P*-value of tiling array analysis was computed using TAS, which uses a Hodges–Lehmann estimator associated with the Wilcoxon rank-sum test to compute the fold enrichment between treatment (cancer) and control (normal) groups. *P*<0.01 was considered to be statistically significant. The *P*-value of expression microarray analysis was determined by one-way ANOVA by comparing triplicate sets of normalised normal and cancer cells. The differential expression of *APOLD1*, *PCDH10*, *RGAG1* and hsa-mir-199a-2 in TGCT patients as determined by qPCR was analysed by two-tailed Student's *t*-test. *P*<0.05 was considered statistically significant.

## Results

### Identification of DMRs in NT2 cells

The pattern of DNA methylation changes substantially when cells become cancerous. To better understand the global change of DNA methylation and the effect on transcription, genome-wide methylation and expression profiling were examined in an *in vitro* pluripotent cell model Ntera2 (NT2), which is an embryonal carcinoma derived from a testicular cancer patient, and in normal testis cells (HT) (Andrews, 1998). Methylated DNA fragments in the genome of each sample were enriched by MeDIP, followed by whole-genome interrogation by hybridising to tiling microarrays that cover the entire non-repetitive human genome.

To highlight the aberrant methylated regions in NT2 cells and to allow downstream processing and analyses, DMR was compiled on the basis of the *P*-value cutoff (*P*<0.01). We identified 22 452 hypermethylated and 12 756 hypomethylated DMRs in the cancer genome. To validate the tiling array results, we randomly selected a number of DMRs at different gene loci including *EBNA1BP2*, *PQLC2*, *HOXC10*, *HOXA7*, *OSR1*, *GAD1*, *ZSWIM2*, and an intergenic region for genomic bisulphite sequencing. The results confirmed the tiling array data and documented it to be a sensitive and reliable tool for detecting DMRs with a *P*-value cutoff at 0.01 (Supplementary Figure 1).

Global DMR differential methylation allowed the analysis of the chromosomal distribution of hypermethylation and hypomethylation, represented by the percentage of the total length of DMRs per 500-kb interval. As anticipated, DMRs were not evenly distributed in the genome. We observed chromosomal regions that were preferentially methylated or demethylated. For example, chromosomes 1p34.3, 1q43–4, 7q36.2–3, 16p13.2 and 21q22.2–3 were intensively hypermethylated, whereas chromosomes 5q13.2, 18q11.2–12.1 and 19q13.31 were more hypomethylated. Some chromosomes, such as 3, 10, 13, 14 and Y, exhibited fewer DMRs (Supplementary Figure 2).

**Figure 1:**
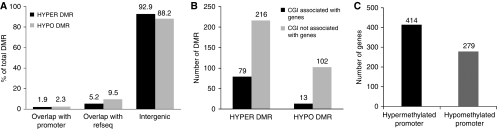
Genome-wide analysis of DMRs. (**A**) Distribution of DMRs. Most of the identified DMRs (88–93%) are mapped to intergenic regions. Promoter DMRs only represent 2% of the total. (**B**) Number of differentially methylated CpG islands that are associated with or without genes. (**C**) Number of differentially methylated promoters.

Aberrant promoter methylation is usually linked to an altered chromosomal state, and thus to transcriptional gene silencing. To determine whether DMRs preferentially occurred in promoters, a genome-wide mapping of DMRs was performed. Intriguingly, most of the human genome DMRs, 92.9% of hypermethylated and 88.2% of hypomethylated DMRs, were mapped to genomic regions without any gene annotation (intergenic). Only 5.2% of hypermethylated and 9.5% of hypomethylated DMRs were mapped to annotated Refseq, including exons and introns. However, a low percentage of DMRs, 1.9% (414) of hypermethylated and 2.3% (279) of hypomethylated DMRs, mapped to promoter regions of known genes (Figure 1A). Thus, we identified a sequence of various chromosomal epigenetic hotspots and many novel DMRs that reside in gene bodies, promoters, CpG islands and intergenic regions.

### Differentially methylated CpG islands and promoters

Although the effect of DNA methylation in intergenic regions is less clear, aberrant methylation in promoter regions has frequently been linked to altered transcriptional activity. About half of the known human gene promoters are associated with CpG islands (Larsen *et al*, 1992). These CpG islands are protected from *de novo* methylation in normal tissues, but often acquire methylation in cancer cells that leads to gene silencing. Among the 35 208 DMRs identified in our study, 410 (295 hypermethylated DMRs and 115 hypomethylated DMRs) overlapped with CpG islands (Supplementary Table 1). However, only 79 (∼27%) hypermethylated CpG islands and 13 (∼13%) hypomethylated CpG islands were associated with gene promoters (Figure 1B). The other differentially methylated CpG islands resided either inside genes or in non-genic regions. For promoter-associated CpG islands, a number of them, including those of *NTF3*, *FGF*, *OSR1*, *HOXA6*, *NPY* and *WT1*, have previously been reported as differentially methylated in other cancer types (Mares *et al*, 2001; Bibikova *et al*, 2006; Oka *et al*, 2006; Houshdaran *et al*, 2007; Illingworth *et al*, 2008). Our study also identified many CpG islands that were not previously shown to be differentially methylated, such as *CXCL5*, *EID1* and *TRHDE*.

Previous studies have suggested that many genes, such as *Oct-4* and *Il2*, lacked CpG islands in their promoters but were regulated by CpG methylation (Bruniquel and Schwartz, 2003; Hattori *et al*, 2004). We undertook a more comprehensive DMR mapping strategy not limited to CpG islands within gene promoters. A total of 693 genes (414 hypermethylated and 279 hypomethylated) were differentially methylated in promoters (Figure 1C and Supplementary Table 2). Compared with previous reports limited to CpG islands, more genes exhibited differential methylation in promoters, which was not associated with CpG islands. Therefore, aberrant promoter methylation is not restricted to CpG islands.

### Variability in the expression of differentially methylated genes

**Figure 2:**
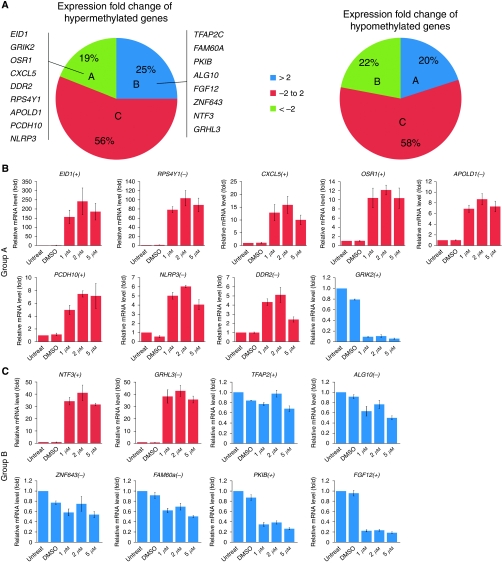
Gene expression of differentially methylated genes. (**A**) Expression of hypermethylated and hypomethylated genes. Genes are divided into three groups on the basis of their expression. Group A: hypermethylated genes (19%) are downregulated (fold change >2), whereas hypomethylated genes (20%) are upregulated (fold change <−2). Group B: hypermethylated genes (25%) are upregulated, whereas hypomethylated genes (22%) are downregulated. Group C: the expression fold change of differentially methylated genes is marginal (fold change between −2 and 2). Nine genes of group A and eight genes of group B are randomly selected and the effect of demethylation is examined, as shown in **B** and **C**. (Panel B) Effect of 5-aza treatment on the expression of nine group A genes. NT2 cancer cells are treated with 1–5 *μ*M 5-aza for 72 h. (Panel C) Effect of 5-aza treatment on the expression of eight group B genes. ‘+’ indicates the promoters that are associated with CpG islands. ‘−’ indicates the absence of CpG islands in the promoters. Error bars indicate s.e.m. of triplicate experiments.

To assess the effect of methylation on transcriptional activity in cancer cells, we carried out a genome-wide analysis of gene expression by microarray. Expression data were then compared with DMR data. On the basis of the relative expression level, genes with differentially methylated promoters could be divided into three groups (Figure 2A). In group A, 19% of hypermethylated genes showed more than two-fold downregulation, whereas 20% of hypomethylated genes showed more than two-fold upregulation. In group B, 25% of hypermethylated genes were upregulated more than two-fold, whereas 22% of hypomethylated genes were downregulated by more than two-fold. In group C, which accounts for 56% of hypermethylated and 58% of hypomethylated genes, the change of expression was marginal (fold change ranged from −2 to 2). The expression of genes in this group seemed to be independent of promoter methylation.

To confirm the effect of CpG methylation on gene expression, we randomly selected eight genes from group A and nine genes from group B, and assessed whether treatment with the demethylating agent 5-aza would restore transcriptional activity. For group A genes, 5-aza treatment restored the expression of eight of the nine selected genes (Figure 2B). For group B, expression of only two of the eight selected genes was restored by 5-aza treatment (Figure 2C). Transcription of most of the genes in group A, but not in group B, suggests a functional role for DNA methylation. The effect of demethylation by 5-aza on gene expression seemed to be independent of the presence of CpG islands.

### Identification of novel aberrantly methylated genes in primary TGCT

**Figure 3:**
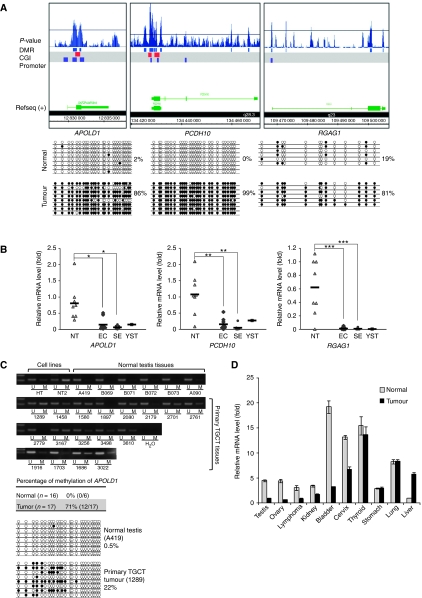
Validation of three hypermethylated candidate genes in primary TGCT samples. (**A**) Hypermethylation of the promoters of *APOLD1*, *PCDH10* and *RGAG1* in NT2 cells. Hypermethylation of these genes is confirmed by genomic bisulphite sequencing. (**B**) Downregulated expression of *APOLD*, *PCDH10* and *RGAG1* in primary TGCT. NT: normal testis (*n*=8); EC: embryonal carcinoma (*n*=9); SE: seminoma (*n*=8); YST (*n*=1): yolk-sac tumour. The mean value of each group is represented by the horizontal bar. ^*^P<0.005; ^**^P<0.05; ^***^P<0.001 by two-tailed Student's *t*-test. (**C**) Promoter hypermethylation of *APOLD1* in primary TGCT. MSP is performed to compare the relative methylation of each patient. In all, 71% of TGCT patients are partially methylated (*n*=17), whereas none of the subjects with a normal testis (*n*=6) are methylated. One case each from the tumour group (1289) and normal group (A419) is selected and confirmed by bisulphite sequencing. U: unmethylated; M: methylated. (**D**) Expression of *APOLD1* in other tumours. RNA samples of each tumour and normal adjacent tissue were isolated from a single individual. Error bars indicate s.e.m. of triplicate experiments.

The testicular embryonal carcinoma NT2 cell is one of the well-studied testicular germ cell neoplasms (Andrews, 1998). On the basis of DMR data, we identified novel hypermethylated candidate genes that might be potential epigenetic markers for TGCT. We selected candidate genes on the basis of the following criteria: first, genes with hypermethylated promoters (Figure 3A); second, expression of genes that are downregulated and in which demethylation by 5-aza restored gene expression (Figure 2B); third, a biological role in testicular cancer was not previously described. The candidate genes were validated in normal testis biopsies and in primary TGCT samples. On the basis of these criteria, we identified three candidate genes, namely *APOLD1*, *PCDH10* and *RGAG1*, for further investigation in the primary TGCT tissue. Promoters of *APOLD1* and *PCDH10* were associated with CpG islands. In contrast, *RGAG1* lacks any CpG island in its promoter region. Hypermethylation of the promoters of these three genes in NT2 cells was confirmed by bisulphite sequencing (Figure 3A). In addition, we examined the methylation status of these genes in another testicular embryonal carcinoma Tera-1. Analogous to NT2 cells, hypermethylation of the three genes in Tera-1 cells was observed (Supplementary Figure 5). In addition, the methylation status of genes in cultured normal testicular cells was similar to that of normal testis tissue, indicating that methylation of these loci was not changed during cell culture (Figure 3A and Supplementary Figure 5). We investigated whether gene expression was altered in a primary TGCT tissue. The expression of these three genes, similar to the results observed in cell culture, was significantly downregulated in both seminoma (*n*=8; *APOLD1*: *P*<0.005; *PCDH10*: *P*<0.05; *RGAG1*: *P*<0.001 by two-tailed Student's *t*-test) and embryonal carcinoma (*n*=9; *APOLD1*: *P*<0.005; *PCDH10*: *P*<0.05; *RGAG1*: *P*<0.0005 by two-tailed Student's *t*-test) and in a case of yolk-sac tumour (*n*=1) compared with normal testicular tissue (*n*=8) (Figure 3B).

Among the candidate genes, hypermethylation of *PCDH10* was implicated in other cancers (Ying *et al*, 2007; Yu *et al*, 2009). The present result supports the role of this putative tumour-suppressor gene in testicular cancer. *APOLD1* is an uncharacterised gene and its biological function is currently unknown. To examine whether aberrant hypermethylation of *APOLD1* is also observed in primary TGCT, the methylation status of the promoter of *APOLD1* was measured by MSP (Figure 3C, upper panel). Hypermethylation of the *APOLD1* promoter was confirmed in 71% (*n*=17) of TGCT specimens. The *APOLD1* promoter was unmethylated in all cases of normal testicular tissue (*n*=6). To validate the result of MSP, a pair of tumour and normal tissues was selected and analysed by bisulphite sequencing (Figure 3C, lower panel). Consistent with the MSP result, bisulphite sequencing showed that this gene was almost unmethylated in normal testicular tissues, but exhibited partial methylation in primary tumours. The observations of hypermethylation and downregulation of *APOLD1* in primary TGCT tissues suggest that DNA methylation has a crucial role in silencing this gene. In a preliminary screening of various primary tumours, the expression of *APOLD1* was downregulated in tumours of not only the testis but also those of the ovary, lymphoma, kidney, bladder and cervix (Figure 3D). RNA samples of each tumour type and the corresponding normal adjacent tissue were collected from a single individual; therefore, the role of *APOLD1* as a tumour-suppressor gene awaits further confirmation with examples of more tumour specimens.

### Differentially methylated ncRNAs and their dysregulation in cancer

**Figure 4:**
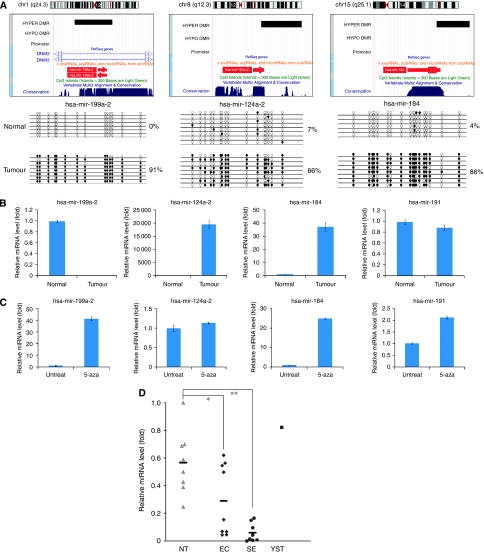
Hypermethylation and differential expression of miRNAs. (**A**) Hypermethylated DMRs at the loci of *hsa-mir-199a-2* (Chr.1q4.3), *hsa-mir-124a-2* (Chr.12q12.3) and *hsa-mir-184* (Chr.15q25.1). *hsa-mir-199a-2* embeds in the intron of *DNM3*, whereas *hsa-mir-124a-2* and *hsa-mir-184* reside in intergenic regions. Hypermethylation of these DMRs in NT2 cells is confirmed by bisulphite sequencing. (**B**) Expression of the three hypermethylated miRNAs as determined by real-time qPCR. *hsa-mir-191* is included as an internal control. Error bars indicate s.e.m. of triplicate experiments. (**C**) Effect of 5-aza treatment on expression of the three hypermethylated miRNAs. (**D**) Dysregulation of *hsa-mir-199a-2* in primary TGCT. The mean value of each group is represented by the horizontal bar. NT: normal testis (*n*=8); EC: embryonal carcinoma (*n*=9); SE: seminoma (*n*=8); YST (*n*=1). ^*^P<0.05; ^**^P<0.00005 by two-tailed Student's *t*-test.

The fact that the majority of DMRs occur in non-repetitive intergenic and intronic regions raises the question of their potential regulatory function. We proposed that intergenic and intronic methylation may have a role in regulating ncRNAs. There are several groups of ncRNAs involved in many cellular processes. microRNA is a class of short ncRNAs that has been known to destabilise or repress translation of mRNA at the post-transcriptional level. To explore the role of intergenic or intronic DMRs, we mapped the identified DMRs to the miRBase Registry. The loci of three miRNAs, namely *hsa-mir-199a-2*, *hsa-mir-124a-2* and *hsa-mir-184*, were found to be linked to hypermethylated DMRs (Figure 4A). Hypermethylation of these three miRNAs in the model culture system (NT2) was confirmed by genomic bisulphite sequencing. To examine the effect of hypermethylation on the expression of miRNA, the level of mature miRNAs in cancer and normal cells was measured by real-time qPCR. Among the three miRNAs, only *hsa-mir-199a-2* was downregulated in cancer cells (741-fold downregulation), whereas *hsa-mir-124a-2* and *hsa-mir-184* showed a 19 562- and 37-fold upregulation, respectively (Figure 4B). Treatment of NT2 cancer cells with 5-aza upregulated the expression of *hsa-mir-199a-2* by 42-fold, indicating that the expression of this miRNA was suppressed by methylation (Figure 4C). Treatment with 5-aza also upregulated the expression of *hsa-mir-184* by 25-fold but had no effect on the expression of *hsa-mir-124a-2*.

*hsa-mir-199a-2* may be a candidate gene that is epigenetically regulated in TGCT. We thus studied its expression in primary TGCT tissue. By real-time qPCR, the expression level of *hsa-mir-199a-2*, as normalised with that of *hsa-mir-191*, was downregulated in embryonal carcinoma (*n*=9; *P*<0.05 by two-tailed Student's *t*-test), and more significantly in seminoma (*n*=8; *P*<0.00005 by two-tailed Student's *t*-test) (Figure 4D).

In addition to miRNA, we also mapped non-genic DMRs to snoRNA-LBME-db, and three snoRNAs, namely *HBII-240*, ACA33 and *ACA8*, were hypomethylated (Supplementary Figure 3A). Quantitation of expression by real-time qPCR analysis of these snoRNAs in cancer and normal cell lines revealed that *HBII-240* and *ACA33* were upregulated by approximately three-fold (Supplementary Figure 3B). In a proportion of primary TGCT tumours, we found that these three snoNRAs were also upregulated as compared with normal testis tissue (Supplementary Figure 4). The specific role of these snoRNAs in testicular germ cell tumourigenesis remains to be elucidated.

## Discussion

Aberrant DNA methylation is common in cancer cells. This study demonstrates a genome-wide approach for identification of differentially methylated genes and ncRNAs using MeDIP-chip in combination with global expression analysis.

CpG island hypermethylation results in changes in chromatin accessibility and seems to repress gene transcription. In our study, although many genes were differentially methylated, only 20% of genes showed an association between hypermethylation and gene repression. The role of DNA methylation on repression of these genes was validated by treatment with 5-aza, which inhibited DNA methylation and restored expression of the genes. We also demonstrated another group of genes that, although hypermethylated in their promoters, were insensitive to demethylation. The existence of methylation-insensitive genes highlights the need to experimentally link epigenetic changes to altered transcriptional activity.

Whole-genome tiling hybridisation allowed us to observe widespread methylation changes. Only a small proportion of DMRs were found in promoters of known genes. A substantial number of DMRs were located in intronic or intergenic regions. Methylation changes in intronic or intergenic regions previously reported have largely been ignored because of failure to investigate transcriptional consequences. The role of intergenic DMRs remains an enigma. They may be a consequence of inappropriate epigenetic change during transformation. They may have a role in maintenance of genomic stability or chromatin condensation (Ahuja *et al*, 1997; Ballestar and Esteller, 2002). Another possible function of intronic and intergenic DMRs is the regulation of genetic elements not identified by conventional algorithms. Many ncRNAs are located in intronic and intergenic regions and their regulation is unknown. In our study, DMRs were mapped to current miRNA and snoRNA databases to explore whether methylation changes occur in regions of ncRNAs. This allowed identification of three hypermethylated miRNAs and three hypomethylated snoRNAs.

Although the three miRNAs were hypermethylated, expression and 5-aza treatment experiments indicated that only *hsa-mir-199a-2* was suppressed by hypermethylation. The unexpected behaviour of *hsa-mir124a-2* and *hsa-mir-184* could probably be explained by the location of the partially methylated regions near the 3′-end of the transcribed locus, whereas the DMR of *hsa-mir-199a-2* covers the 5′ upstream and transcribed locus. Studies of cancers report that miRNA dysregulation is often associated with tumour progression or metastasis, probably a consequence of post-transcriptional silencing of target oncogenes or tumour-suppressor genes (Mendell, 2005; Zhang *et al*, 2007). This study implicates methylation as one of the causes.

Small nucleolar RNAs are another group of ncRNAs that guide modification of rRNAs or spliceosomal RNAs. These conserved small RNA regulators modify alternative splicing of many transcripts (Bachellerie *et al*, 2002). In this study, the identification of hypomethylation and enhanced expression of the three snoRNAs suggest a potential relationship between cancer and dysregulation of snoRNAs.

An *in vitro* cell culture system was exploited in this study because of the ease of its manipulation. We recognise that methylation changes in these cultured cells may not reflect *in vivo* changes. Despite this, we found a number of differentially methylated genes in the culture system, which were concordant with those of tissue samples. Three hypermethylated genes, *PCDH10*, *APOLD1* and *RGAG1*, were investigated as examples. These genes were silenced in primary TGCTs and their expression was restored upon demethylation. *PCDH10* encodes a membrane protein for cell adhesion. It has been implicated to be a tumour-suppressor gene in studies on nasopharyngeal, oesophageal, breast, colorectal, cervical, lung and hepatocellular carcinoma cell lines. Expression of *PCDH10* in these cell lines was suppressed by DNA hypermethylation (Ying *et al*, 2006). Interestingly, it has also been identified as one of the deleted loci in patients with autism (Morrow *et al*, 2008). Before this study, *RGAG1*and *APOLD1* were not known to be epigenetically silenced in cancers. *RGAG1* (also known as *MART9*) is an X-linked retrotransposon-derived neogene of unknown function (Brandt *et al*, 2005). Expressed sequence tags of *RGAG1* were found predominantly in the testis, suggesting that this retrogene might be important in germ cell development. *APOLD1* is another uncharacterised gene identified in this study. Its open reading frame encodes an apolipoprotein-L domain-containing protein, the function of which is unknown. Remarkably, *APOLD1* is located in 12p13.1, a TGCT susceptibility locus previously identified by genetic linkage analysis (Crockford *et al*, 2006). Although genetic susceptibility loci in this gene have not been identified, the coincidence of an epigenetically silenced gene in this locus may provide a new insight into interactions between genetic and epigenetic factors.

In summary, this study provides comprehensive data for identification of both protein-coding genes and ncRNAs that are epigenetically regulated by DNA methylation. Methylation occurs in promoters and CpG islands, as well as in intragenic and intergenic regions. Only a subset of hypermethylated genes is directly regulated by DNA methylation. We also demonstrated dysregulation of three candidate genes and a miRNA in primary TGCTs. Two of the genes, *APOLD1* and *RGAG1*, are novel genes, the biological function of which needs further investigation. *hsa-mir-199a-2* is another developmentally regulated miRNA that is implicated in cancer invasion (Migliore *et al*, 2008).

We now use the MeDIP-chip for identifying aberrant methylation in malignant TGCT patient samples targeting for DMRs specific for cancer metastasis. Meanwhile, we identified potential targets of *hsa-mir-199a-2* that might be associated with cancer invasion in TGCT (data unpublished).
