# Supplementary figures and images for: Genome-wide DNA methylation profiling reveals novel epigenetically regulated genes and non-coding RNAs in human testicular cancer
Source: Br J Cancer. 2010 Jan 5;102(2):419–27. doi: 10.1038/sj.bjc.6605505 (PMC2816664; doi:10.1038/sj.bjc.6605505)

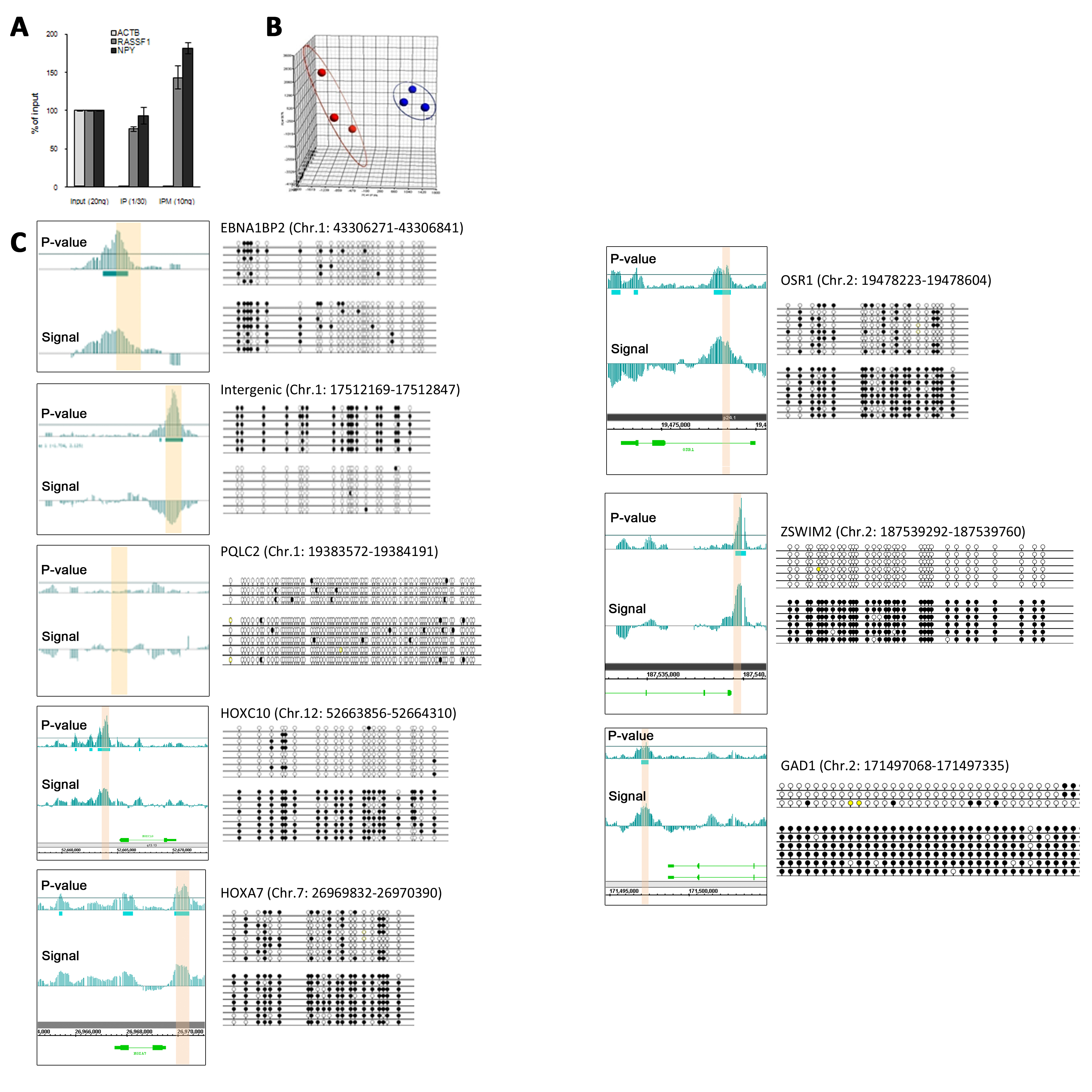

Supplement: Supplementary Figure 1 [file 6605505x1.tif]

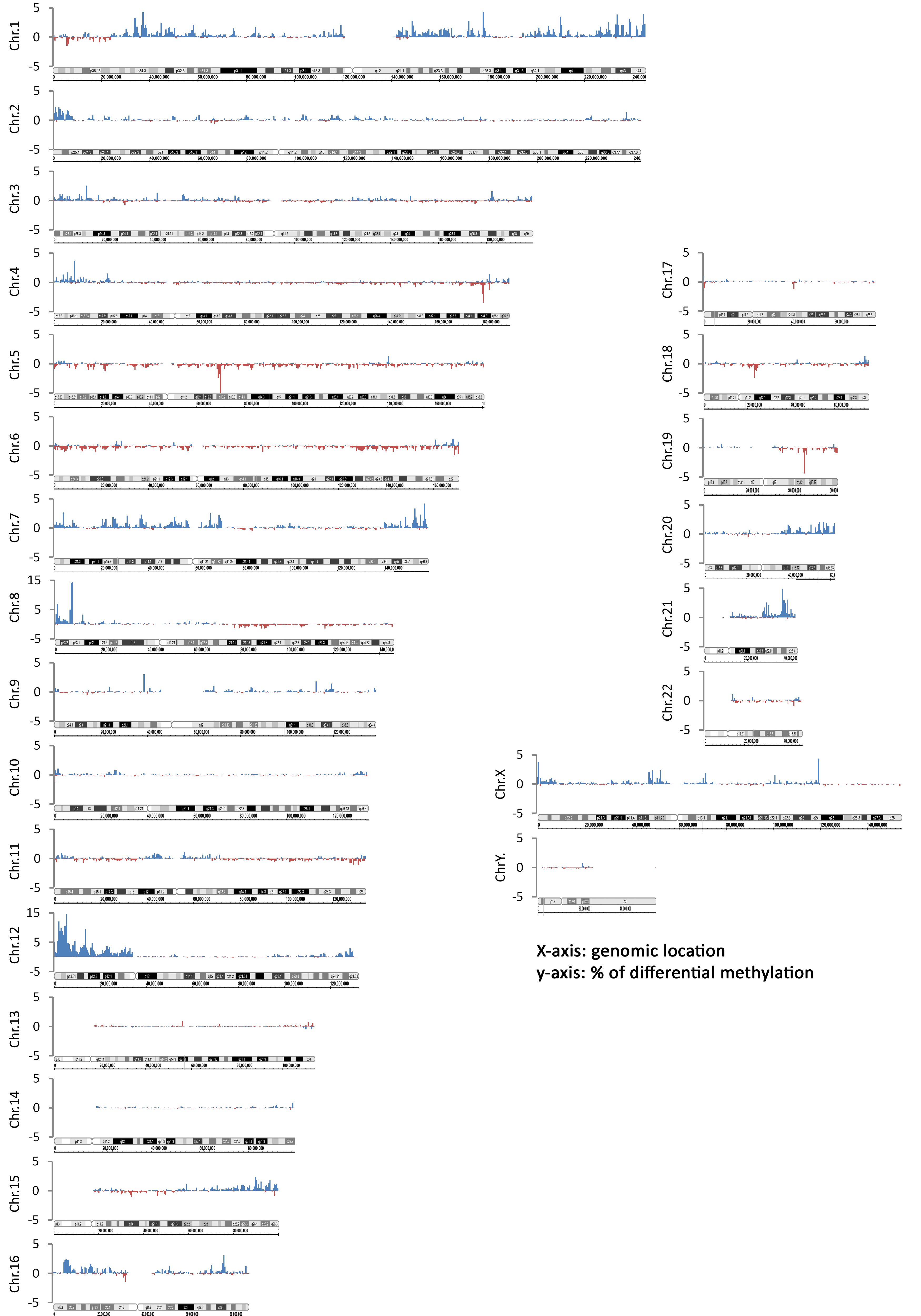

Supplement: Supplementary Figure 2 [file 6605505x2.tif]

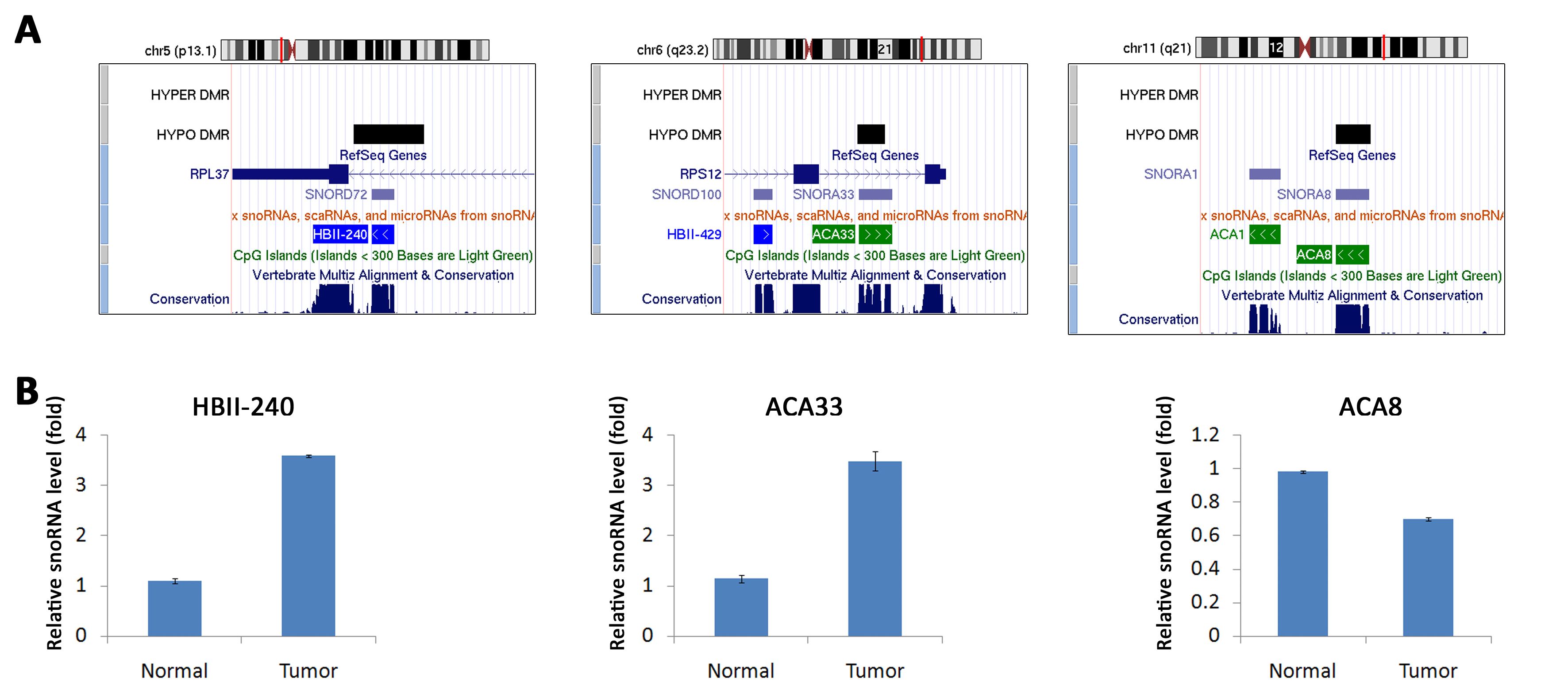

Supplement: Supplementary Figure 3 [file 6605505x3.tif]

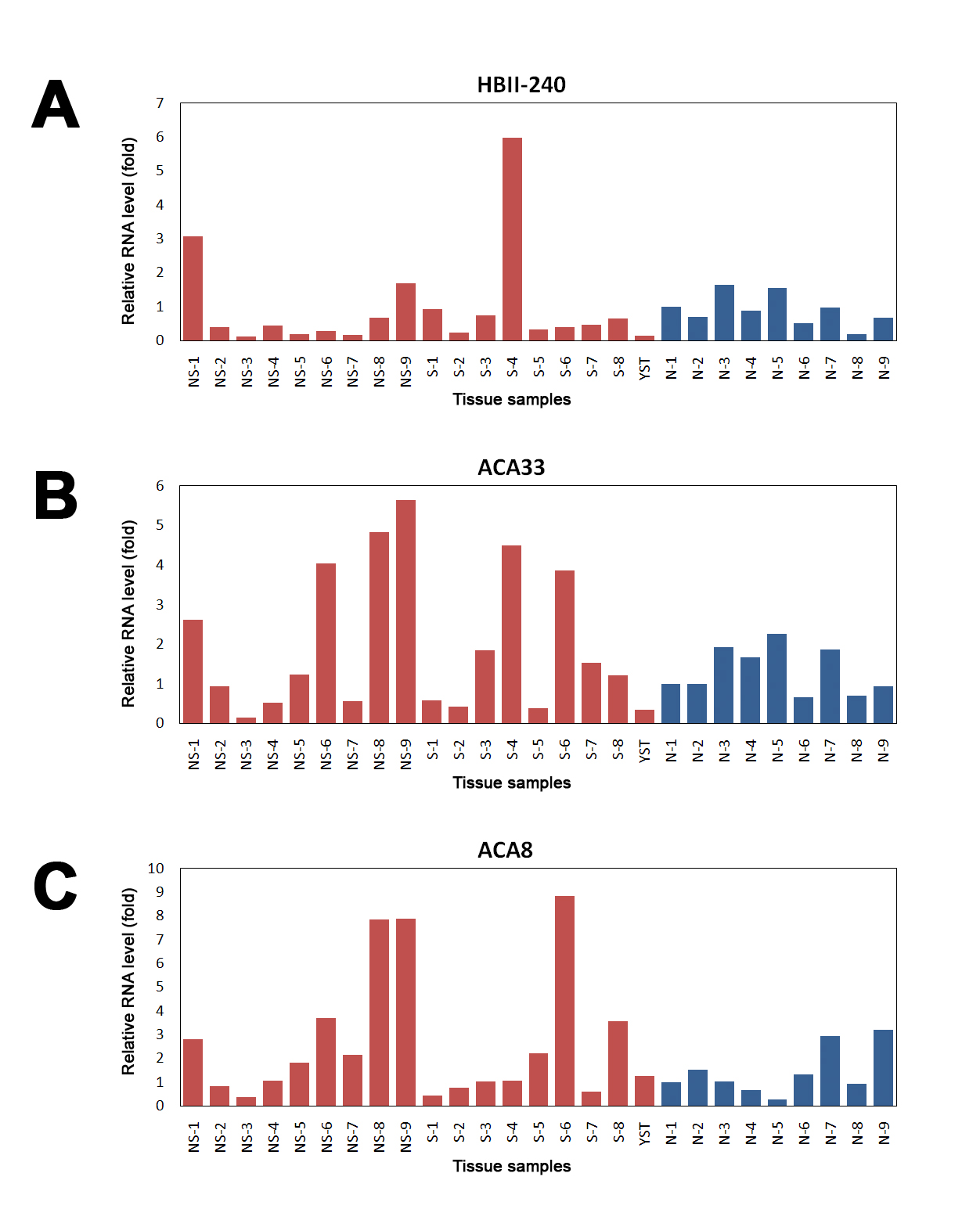

Supplement: Supplementary Figure 4 [file 6605505x4.tif]

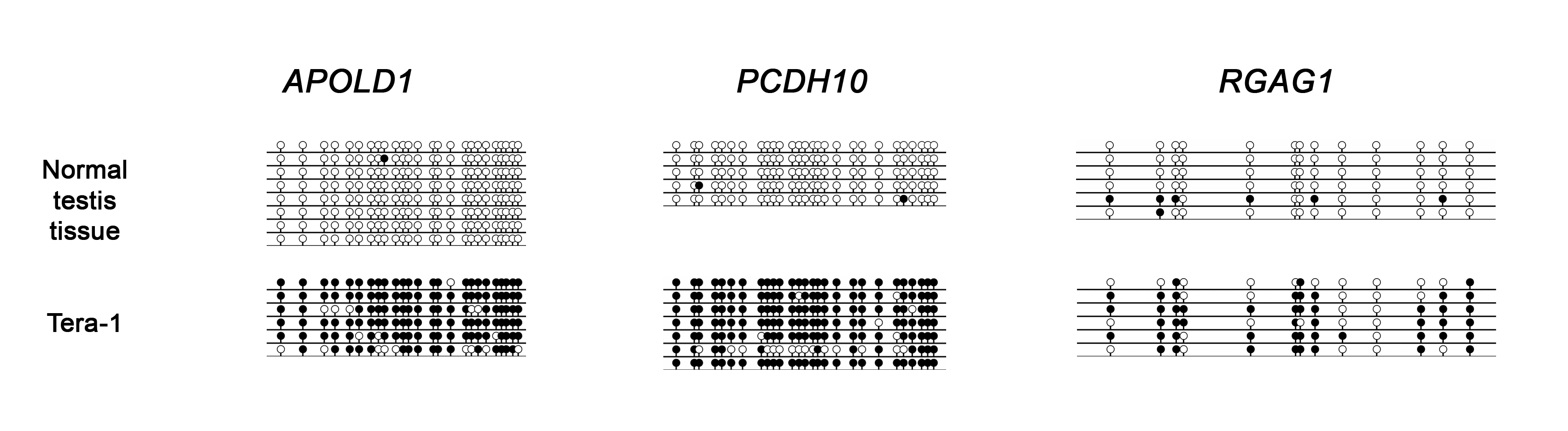

Supplement: Supplementary Figure 5 [file 6605505x5.tif]
