# Supplementary material for: Genome-wide DNA methylation profiling reveals novel epigenetically regulated genes and non-coding RNAs in human testicular cancer
Source: Br J Cancer. 2010 Jan 5;102(2):419–27. doi: 10.1038/sj.bjc.6605505 (PMC2816664; doi:10.1038/sj.bjc.6605505)
Supplement: Supplementary Information [file 6605505x10.doc]

**Supplementary figure legends**

**Supplementary Figure 1**. Validation of MeDIP-Chip result and confirmation of DMRs by bisulfite sequencing. (**A**) Validation of MeDIP. Real-time qPCR analysis of *ACTB*, *RASSF1* and *NPY* on immunoprecipitated DNA (IP) and immunoprecipitated DNA with PCR amplification (IPM). Input DNA serves as background control. *RASSF1* and *NPY* are two positive methylation controls while *ACTB* is a methylation negative control. Error bars indicate s.e.m. of triplicate experiments. (**B**) Principal Components Analysis (PCA) of triplicate sets of array hybridization of the normal (red) and cancer (blue) cell lines. The distinct distribution between the two groups indicated that global methylation is different. (**C**) Different loci from different chromosomes (*EBNA1BP2* and *PQLC2* on chromosome 1, *HOXC10* on chromosome 12, *HOXA7* on chromosome 7, *OSR1*, *GAD1*, and *ZSWIM2* on chromosome 2, and an intergenic region on chromosome 1) as printed in different tiling array chips are selected and differential methylation is confirmed by bisulfite sequencing.

**Supplementary Figure 2**. Distribution of hypermethylation (blue peaks) and hypomethylation (red peaks) in all chromosomes. Differential methylation is represented as percentage of the total length of hypermethylated or hypomethylated DMRs in a 500 kb interval and plotted across the genome.

**Supplementary Figure 3**. Hypomethylation of snoRNAs. (**A**) Three conserved snoRNAs, HBII-240, ACA33 and ACA8, are hypomethylated. HBII-240 and ACA33 reside in the introns of *RPL37* and *RPS12* respectively, while ACA8 is found in intergenic region. (**B**) Real time qPCR analysis on the expression of the 3 snoRNAs. Both ACA33 and HBII-240 are upregulated by 3 fold in NT2 cancer cells. Error bars indicate s.e.m. of triplicate experiments.

**Supplementary Figure 4**. Real time qPCR analysis on the expression of the 3 snoRNAs in primary TGCT. (**A**) HBII-240. (**B**) ACA33. (**C**) ACA8. NS: non-seminoma; S: seminoma; YST: yolk sac tumor; N: normal testis.

**Supplementary Figure 5.** Bisulfite sequencing of the 3 candidate genes (*APOLD1*, *PCDH10* and *RGAG1*) in normal testis tissue and another human testicular embryonal carcinoma cell line Tera-1.

**Supplementary tables**

**Supplementary Table 1**. Differentially methylated CpG islands associated with genes and expression data of the corresponding genes.

**Supplementary Table 2**. Differentially methylated promoters with expression data of the corresponding genes.

**Supplementary Table 3**. Validation of microarray expression data by real-time qPCR.

**Supplementary Table 4**. Primer sequences used in this study.
